# Association between serum anion gap and all-cause mortality in critically ill patients with diabetic kidney disease: Analysis of the MIMIC-IV database

**DOI:** 10.1371/journal.pone.0329269

**Published:** 2025-08-01

**Authors:** Yu-Nan Han, Cheng-Yue Xiong, Yong-Xiang Wang, Jun-Li Yuan, Lin Li, Zui-Xuan Xiao

**Affiliations:** 1 Department of Endocrinology, The First Affiliated Hospital of Yangtze University, Jingzhou, Hubei, China; 2 Department of Medicine, Yangtze University, Jingzhou, Hubei, China; 3 Yueyang Central Hospital, Yueyang, Hunan, China; University of Milan: Universita degli Studi di Milano, ITALY

## Abstract

**Background:**

Diabetic kidney disease (DKD) is one of the most common diabetic complications and is closely related to metabolic acidosis. The serum anion gap (AG), an important index of acid-base balance, may reflect disturbed metabolism and be correlated with increased mortality risk. However, the role of AG in all-cause mortality risk in individuals suffering from severe DKD is not yet clear.

**Methods:**

In this study, patients identified with a diagnosis of severe DKD utilizing MIMIC-IV database were determined. They were subsequently divided into four quartiles based on their serum AG levels. The findings consisted of in-hospital deaths and ICU deaths. The relationship between serum AG levels of severe DKD patients and clinical outcomes was elucidated using Cox proportional hazards regression analysis and RCS analysis.

**Results:**

In total, 1,716 patients (66.43% male) received treatment, with in-hospital and ICU mortality rates reaching 16.43% and 13.17%, respectively. Multivariate Cox regression analysis revealed that elevated serum AG levels were remarkably linked to all-cause mortalities. When adjusted for confounders, elevated serum AG levels correlated notably with in-hospital mortality [HR = 1.09(95%CI:1.07,1.11)P < 0.0001] and ICU mortality [HR = 1.10(95%CI:1.07,1.12)P < 0.0001]. RCS analysis showed that serum AG levels were positively correlated in a linear fashion with all-cause death risk.

**Conclusion:**

Serum AG shows a significant positive correlation with all-cause mortalities in hospitals and ICU settings among patients with severe DKD. This suggests that serum AG could serve as a potential indication for recognizing DKD individuals with an increased overall risk of all-cause death.

## Introduction

Diabetes mellitus (DM) continues to pose a major threat for human well-being as one of the major medical challenges in this century. According to the 10th Edition of the International Diabetes Federation (IDF) Diabetes Atlas (2021 data), approximately 537 million adults (aged 20–79 years) worldwide have diabetes, accounting for 10.5% of the adult population; this figure is projected to rise to 783 million (12.2%) by 2045 [1]. Diabetic kidney disease (DKD) is a common microvascular complication of diabetes, defined by persistent albuminuria and/or a reduced glomerular filtration rate (GFR) in individuals with diabetes [2]. As the population of patients with DM expands, the concomitant rise in the prevalence of DKD is observed [3,4]. Diabetic kidney disease (DKD) has become a leading cause of end-stage renal disease (ESRD) [5]. This has led to decreased quality of life, decreased survival time for patients, and imposed substantial social and economic burdens [6].

Research has demonstrated that the body’s acid-base balance significantly influences the onset and progression of DKD. Diabetic patients are often at risk of metabolic acidosis, particularly as renal function declines, exacerbating kidney damage and being associated with all-cause mortality. Metabolic acidosis is highly prevalent in intensive care units (ICUs) and is typically present upon admission [7]. Studies indicate that metabolic acidosis accelerates the progression of kidney disease through various mechanisms, including promoting inflammatory responses, increasing oxidative stress, and stimulating fibrosis [8,9]. This phenomenon is detrimental to renal outcome in chronic kidney disease (CKD) patients. Furthermore, its pivotal role in the onset and progression of kidney injury associated with DM has been demonstrated. Further research has identified that local lactic acidosis, induced by enhanced lactate dehydrogenase A (LDHA) activity in diabetic patients, is a key driver in the progression of DKD. Lactic acidosis accelerates renal function deterioration by exacerbating renal fibrosis and mitochondrial dysfunction. Inhibition of LDHA activity significantly improves renal fibrosis and functional damage, suggesting that local lactic acidosis may serve as a potential intervention target for DKD [10]. Additionally, another study has shown that correcting metabolic acidosis by reducing dietary acid load and appropriately supplementing alkaline agents, such as bicarbonate, can slow renal function deterioration, emphasizing the crucial role of acid–base balance in the management of kidney disease [11].

The serum anion gap (AG), representing the difference among not-measured positive and negative ions of blood, is an important indicator for determining the type of metabolic acidosis and assessing acid-base status [12–14]. A substantial body of research has demonstrated a linear association between elevated AG levels and higher mortality rate in ICU patients afflicted with conditions such as cerebral infarction [15], aortic aneurysm [16], acute kidney injury (AKI) [17], acute pancreatitis [18], and sepsis [19].

Despite the growing interest in DKD, an in-depth examination of associations of AG with all-cause death rates in patients diagnosed with severe DKD has yet to be conducted. Therefore, this study aims to further explore the relationship between AG and all-cause mortality in critically ill DKD patients by analyzing the Medical Information Mart for Intensive Care III (MIMIC-IV).

## Materials

### Source of data

This retrospective research analyzes health-associated statistics acquired through MIMIC-IV database (version 3.1), a vast and consolidated repository created and maintained by MIT Laboratory for Computational Physiology. This database contains a substantial number of top-quality healthcare accounts of ICU patients at Beth Israel Deaconess Medical Center between 2008 and 2022 [20]. An author (YuNan Han) satisfied the National Institutes of Health’s web-based course “Protecting Human Research Participants” (Recode ID: 65,640,325), fulfilling the database access requirements and assuming responsibility for data extraction. To protect patient privacy, the data were de-identified. Consequently, the Beth Israel Deaconess Medical Center Ethics Committee waived the requirement for patient informed consent. The study’s description adheres to the Strengthening the Reporting of Observational Studies in Epidemiology (STROBE) statement and complies with the Declaration of Helsinki and the NIH’s “Protecting Human Research Participants” course.

### Study population

**Fig 1:**
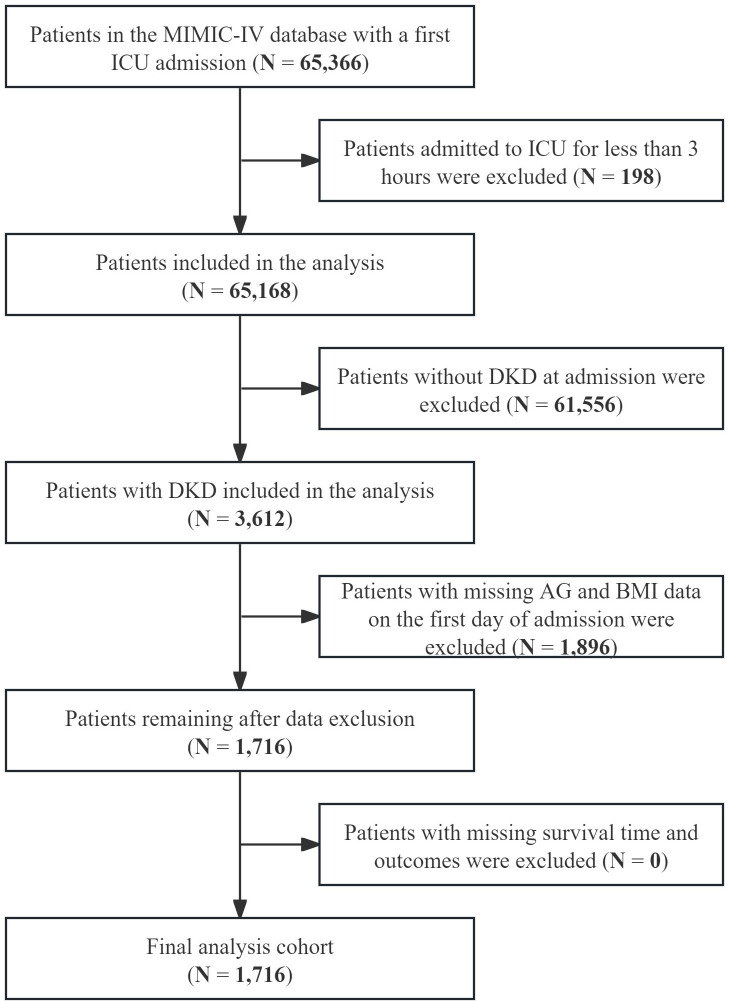
Flowchart of the selection strategy.

In this study, diabetic kidney disease (DKD) was diagnosed on the basis of the International Classification of Diseases (ICD) coding standards. Specifically, ICD-9 codes 250.40–250.43 and ICD-10 codes E08.2, E08.21, E08.22, E08.29; E10.2, E10.21, E10.22, E10.29; E11.2, E11.21, E11.22, E11.29; and E13.2, E13.21, E13.22, E13.29 were utilized. The study applied the following exclusion criteria: (1) age less than 18 years at first admission; (2) ICU stays lasting less than three hours; (3) multiple ICU admissions for DKD with data retained for the initial admission only; and (4) incomplete data on AG, BMI, survival time, or patient outcomes. Ultimately, 1,716 DKD patients were enrolled in this analysis (Fig 1).

### Data collection

Information was extracted with PostgreSQL (version 16.6) and Navicat Premium (version 17) software by executing Structured Query Language (SQL) commands. Potential parameters were extracted in six major groupings: (1) demographics, including age, sex, weight and body mass index; (2) vital signs, such as blood pressure(BP), body temperature(TP), heart rate(HR), and oxygen saturation(SPO_2_); (3) comorbid conditions, such as hypertension, congestive heart failure, moderate to severe liver disease, cerebrovascular disease, and metastatic solid tumors; (4) lab markers, consisting of red blood cells (RBC), white blood cells (WBC), platelets (PLT), red cell distribution width (RDW), hemoglobin (HB), sodium, potassium, creatinine(Cr), blood urea nitrogen (BUN), bicarbonate(HCO3^-^), oxygen saturation(SPO_2_), partial pressure of carbon dioxide(PCO_2_), pH, lactate(LAC) and glucose (GLU); (5) severity scores at admission, ranging from Acute Physiology Score III (APSIII), Simplified Acute Physiology Score II (SAPS-II), Sepsis-related Organ Failure Assessment score (SOFA) [21,22], and the Glasgow Coma Scale (GCS); and (6) commonly used ICU medications, such as insulin. Serum anion gap (AG) values were designated as both the study variable and the primary exposure factor. For each lab variable and disease severity score, data were taken from patients admitted to ICU within 24 hours of their admission.

To avoid potential bias, variables with more than 20% missing values were excluded. Variables with less than 20% missing data were subjected to multiple imputation using the R package missForest, which employs a random forest algorithm trained on the other non‐missing variables [23].

### Clinical outcomes

The primary endpoint was defined as in-hospital all-cause mortality, while the secondary endpoint was defined as mortality occurring in the intensive care unit.

### Statistical analysis

Continuous variables were summarized as mean ± standard deviation or median (interquartile range), as appropriate, and categorical variables were presented as percentages. Normality of continuous parameters was assessed by the Shapiro–Wilk test. For normally distributed variables, between-group comparisons were performed using the Student’s t-test or one-way analysis of variance; for non-normally distributed variables, the Mann–Whitney U-test or Kruskal–Wallis test was applied. Serum AG was first analyzed as a continuous variable and subsequently classified into quartiles, with the lowest quartile (Q1) serving as the reference group. Survival probabilities across serum AG quartiles were estimated by Kaplan–Meier (KM) analysis and compared using the log-rank test. The predictive performance of serum AG was evaluated and compared with other acid-base balance indicators, including bicarbonate, PCO₂, pH, and lactate, using receiver operating characteristic (ROC) curve analysis. The area under the curve (AUC) was calculated for each indicator to assess their diagnostic accuracy in predicting the outcome of interest. To identify factors linked to all-cause mortality risk, binary logistic regression analysis was conducted. Multivariable weighted Cox regression patterns and restrictive cubic splines (RCS) were subsequently implemented to compute hazard ratios (HRs) and 95% credibility intervals (CIs), with appropriate adjustments to models. Clinically and prognostically relevant variables were incorporated into the multivariable models as follows: Model 1: Unadjusted; Model 2: Adjusted for sex, age, BMI; Model 3: Adjusted for sex, age, BMI, hypertension, congestive heart failure, moderate to severe liver disease, cerebrovascular disease, metastatic solid tumor, PLT, RBC, WBC, BUN, Sodium, Potassium, GLU. Subsequently, we conducted subgroup analyses to examine the potential modifying effects of key demographic and clinical variables on the relationship between serum AG and mortality outcomes, with all covariates (except those used for stratification) adjusted in the models. Stratification was performed based on sex, age (<60 years/ ≥ 60 years), BMI (<25 kg/m²/ ≥ 25 kg/m²), congestive heart failure, cerebrovascular disease, moderate to severe liver disease, metastatic solid tumor, and insulin use. Interaction between serum AG and these variables was assessed by including multiplicative interaction terms (AG × covariates) to the Cox proportionate hazards regression model. For determining if the relation that exists between serum AG and mortality varies across different subgroups. Variance analysis was utilized to test the significance of interactions, with a two-sided P < 0.05 deemed statistical important. All strategic analyses are carried out with R software (version 4.4.1).

## Result

A total of 1,716 patients with severe diabetic kidney disease (DKD) were enrolled in this study, of whom 33.57% were female and 66.43% were male. Mean age was ranged from 71.18 years (IQR: 63.28–78.52 years). The median serum AG for all patients was 15 (IQR: 12–19). In-hospital and ICU mortalities respectively amounted to 16.43% and 13.17% (Table 1).

**Table 1:** Characteristics and outcomes of participants categorized by AG.

Variable	Total (n = 1716)	Q1 (n = 468)	Q2 (n = 461)	Q3 (n = 447)	Q4 (n = 340)	Statistic	P value
Sex, n(%)						4.34	0.23
Female	576(33.57)	142(30.34)	151(32.75)	161(36.02)	122(35.88)		
Male	1140(66.43)	326(69.66)	310(67.25)	286(63.98)	218(64.12)		
Age, years,(median [IQR])	71.18(63.28,78.52)	71.69(64.39,79.70)	71.38(63.33,78.41)	70.61(63.28,77.44)	70.88(60.61,78.64)	4.64	0.20
BMI, kg/m^2^, n(median [IQR])	29.77(25.76,34.90)	30.22(26.01,34.81)	30.57(26.18,35.75)	29.07(25.49,34.55)	29.02(25.41,34.80)	8.54	0.04
Weight, kg, (median [IQR])	86.03(72.50,101.93)	87.75(74.71,104.04)	87.20(73.65,102.25)	83.30(71.37,99.08)	84.35(71.14,100.93)	7.71	0.05
Vital signs SBP, mmhg, n(median [IQR])	115.15(107.50,126.08)	113.85(108.67,121.67)	117.74(108.22,130.00)	116.16(107.92,127.30)	113.27(103.10,123.30)	24.47	<0.0001
DBP, mmhg, n(median [IQR])	57.75(52.21,64.88)	56.51(51.71,62.32)	57.48(52.25,65.03)	59.00(52.78,66.13)	58.48(52.21,66.83)	11.59	<0.01
TP, (median [IQR])	36.74(36.53,36.98)	36.70(36.49,36.93)	36.75(36.57,37.00)	36.77(36.55,37.03)	36.72(36.48,36.98)	15.15	<0.01
SPO_2_, (median [IQR])	97.61(96.14,98.80)	97.88(96.65,98.88)	97.58(96.13,98.82)	97.35(95.86,98.67)	97.45(96.04,98.84)	18.09	<0.001
HR, (median [IQR])	80.00(71.95,90.07)	78.02(71.84,85.64)	78.58(70.86,88.85)	80.45(71.17,90.83)	85.00(74.39,98.26)	48.63	<0.0001
PCO_2_, (median [IQR])	45.00(40.00,50.05)	47.00(43.00,51.05)	46.00(41.00,51.40)	44.00(39.00,50.00)	41.00(36.00,47.00)	115.82	<0.0001
PH, (median [IQR])	7.41(7.38,7.44)	7.42(7.39,7.45)	7.41(7.38,7.44)	7.41(7.37,7.44)	7.40(7.35,7.43)	37.76	<0.0001
LAC, (median [IQR])	2.53(1.90,3.60)	2.43(1.86,3.11)	2.40(1.90,3.20)	2.48(1.80,3.50)	3.40(2.08,5.73)	76.06	<0.0001
Medical scores Sofa, (median [IQR])	6.00(4.00,9.00)	5.00(4.00,7.00)	6.00(3.00,8.00)	7.00(4.00,9.00)	8.00 (6.00,11.00)	156.17	<0.0001
ApsⅢ, (median [IQR])	51.00(39.00,65.00)	42.00(33.00,54.00)	47.00(38.00,60.00)	54.00(43.00,66.00)	65.00(52.00,83.00)	263.58	<0.0001
SapsⅡ, (median [IQR])	42.00(35.00,52.00)	40.00(33.00,47.00)	40.00(33.00,48.00)	43.00(35.00,53.00)	51.00(40.00,63.00)	139.48	<0.0001
GCS, (median [IQR])	15.00(14.00,15.00)	15.00(14.00,15.00)	15.00(14.00,15.00)	15.00(14.00,15.00)	15.00(14.00,15.00)	3.89	0.27
Commorbidities Hypertension, n(%)						24.31	<0.0001
No	1625(94.70)	437(93.38)	421(91.32)	434(97.09)	333(97.94)		
Yes	91(5.30)	31(6.62)	40(8.68)	13(2.91)	7(2.06)		
Congestive Heart failure, n(%)						36.58	<0.0001
No	743(43.30)	249(53.21)	209(45.34)	155(34.68)	130(38.24)		
Yes	973(56.70)	219(46.79)	252(54.66)	292(65.32)	210(61.76)		
Moderate/severe Liver disease, n(%)						15.25	<0.01
No	1633(95.16)	458(97.86)	442(95.88)	418(93.51)	315(92.65)		
Yes	83(4.84)	10(2.14)	19(4.12)	29(6.49)	25(7.35)		
Cerebrovascular disease, n(%)						0.83	0.84
No	1442(84.03)	393(83.97)	382(82.86)	380(85.01)	287(84.41)		
Yes	274(15.97)	75(16.03)	79(17.14)	67(14.99)	53(15.59)		
Metastatic Solid tumor, n(%)						4.98	0.17
No	1675(97.61)	460(98.29)	445(96.53)	440(98.43)	330(97.06)		
Yes	41(2.39)	8(1.71)	16(3.47)	7(1.57)	10(2.94)		
Medications Insulin, n(%)						6.15	0.10
No	65(3.79)	11(2.35)	15(3.25)	21(4.70)	18(5.29)		
Yes	1651(96.21)	457(97.65)	446(96.75)	426(95.30)	322(94.71)		
Laboratory tests							
HB, g/dL, (median [IQR])	9.70(8.70,10.80)	9.63(8.78,10.80)	9.80(8.90,11.10)	9.60(8.60,10.80)	9.60(8.58,10.60)	9.20	0.03
PLT, K/uL, (median [IQR])	181.00(137.00,235.00)	166.00(133.00,217.00)	187.00(146.00,239.00)	189.00(140.00,243.50)	190.00(125.00,246.00)	16.16	<0.01
RBC, m/uL, (median [IQR])	3.31(2.94,3.74)	3.29(2.95,3.72)	3.35(3.00,3.81)	3.31(2.92,3.71)	3.26(2.88,3.67)	6.44	0.09
RDW, fL, (median [IQR])	15.10(14.10,16.70)	14.50(13.60,15.83)	14.86(14.00,16.30)	15.50(14.40,17.10)	16.06(14.80,17.90)	142.43	<0.0001
WBC, K/uL, (median [IQR])	12.10(9.20,16.20)	12.10(9.39,15.70)	11.80(9.18,15.70)	12.00(8.90,15.70)	13.15(9.50,18.34)	11.33	0.01
AG, mEq/L, (median [IQR])	15.00(12.00,19.00)	11.00(9.00,12.00)	14.00(13.00,15.00)	17.00(16.00,18.00)	23.00(21.00,25.00)	1610.95	<0.0001
Bic, mEq/L, (median [IQR])	22.00(20.00,24.00)	23.00(21.00,25.00)	22.00(21.00,25.00)	22.00(19.00,25.00)	20.00(17.00,23.00)	134.95	<0.0001
BUN, mg/dL, (median [IQR])	37.00(24.00,58.25)	25.00(18.00,36.00)	33.00(24.00,49.00)	47.00(32.00,67.00)	63.00(41.00,89.00)	427.26	<0.0001
Cr, mg/dL, (median [IQR])	2.10(1.40,3.80)	1.45(1.10,1.90)	1.80(1.40,2.80)	2.50(1.70,4.00)	4.60(2.70,7.10)	493.38	<0.0001
GLU, mg/dL, (median [IQR])	152.00(117.00,213.00)	128.10(105.00,158.25)	147.00(114.00,198.00)	173.00(131.50,249.00)	191.50(141.75,277.25)	195.16	<0.0001
Sodium, mEq/L, (median [IQR])	138.00(136.00,141.00)	138.00(136.00,140.00)	139.00(137.00,141.00)	138.00(136.00,141.00)	138.00(135.00,141.00)	13.73	<0.01
Potassium, mEq/L, (median [IQR])	4.60(4.20,5.10)	4.60(4.28,5.00)	4.50(4.10,5.00)	4.50(4.10,5.00)	4.80(4.30,5.40)	24.96	<0.0001
Events Los hospital days	10.11(6.36,17.38)	9.53(6.68,14.88)	10.78(6.24,16.14)	9.98(6.21,18.35)	11.09(5.77,21.01)	3.06	0.38
Los ICU day	3.12(1.48,6.22)	2.27(1.30,4.26)	2.88(1.42,6.10)	3.77(1.83,7.10)	4.05(1.96,7.96)	53.49	<0.0001
Hospital mortality						111.77	<0.0001
Alive	1434(83.57)	439(93.80)	403(87.42)	365(81.66)	227(66.76)		
Death	282(16.43)	29(6.20)	58(12.58)	82(18.34)	113(33.24)		
ICU mortality						103.77	<0.0001
Alive	1490(86.83)	448(95.73)	414(89.80)	384(85.91)	244(71.76)		
Death	226(13.17)	20(4.27)	47(10.20)	63(14.09)	96(28.24)		

Abbreviation: AG, Anion Gap; BMI, Body Mass Index; SBP, Systolic Blood Pressure; DBP, Diastolic Blood Pressure; TP, Temperature; HR, Heart Rate; LAC, Lactic Acid; SOFA, Sequential Organ Failure Assessment; APS3, Acute Physiology Score III; SAPSII, Simplified Acute Physiology Score II; GCS, Glasgow Coma Scale; HB, Hemoglobin; PLT, Platelet; RBC, Red Blood Cell; RDW, Red Cell Distribution Width; WBC, White Blood Cell; Bic, Bicarbonate; BUN, Blood Urea Nitrogen; Cr, Creatinine; GLU, Glucose.

### Baseline characteristics

Table 1 presents the baseline features of critically ill DKD individuals categorized by serum AG quartiles. Participants on admission are divided into 4 cohorts in accordance with their serum AG levels, with median AG values of 11 (IQR: 9–12), 14 (IQR: 13–15), 17 (IQR: 16–18), and 23 (IQR: 21–25) for each quartile, respectively. Patients in the highest quartile of serum AG levels are typically characterized by elevated heart rates, more severe illness severity scores upon admission, as well as higher RDW, BUN, Cr, and GLU levels. When compared to those in lower serum AG quartiles, patients in the higher quartiles experience longer ICU stays (2.27 days vs. 2.88 days vs. 3.77 days vs. 4.05 days, P < 0.0001), higher inpatient mortality rates (6.20% vs. 12.58% vs. 18.34% vs. 33.24%, P < 0.0001), and increased ICU mortality rates (4.27% vs. 10.20% vs. 14.09% vs. 28.24%, P < 0.0001). Considering a stronger correlation observed among the fourth quartile and all-cause mortalities, further differences were analyzed in Q4 and Q1–Q3, and the results indicated that different grouping methods yielded similar outcomes (S1 Table).

Baseline characteristics during hospitalization between the survival and death groups are summarized in S2 Table. Patients in the death group tend to be older, predominantly male, with higher severity scores, heart rates, and lactate levels, in addition to elevated RDW, WBC, BUN, creatinine, and GLU levels. The serum AG level exhibited a significant increase in dead subjects in comparison with surviving subjects (18 vs. 15, P < 0.05). S1 Fig displays the distribution of serum AG stratified by all-causation in-hospital and ICU death status.

### Primary outcomes

**Fig 2:**
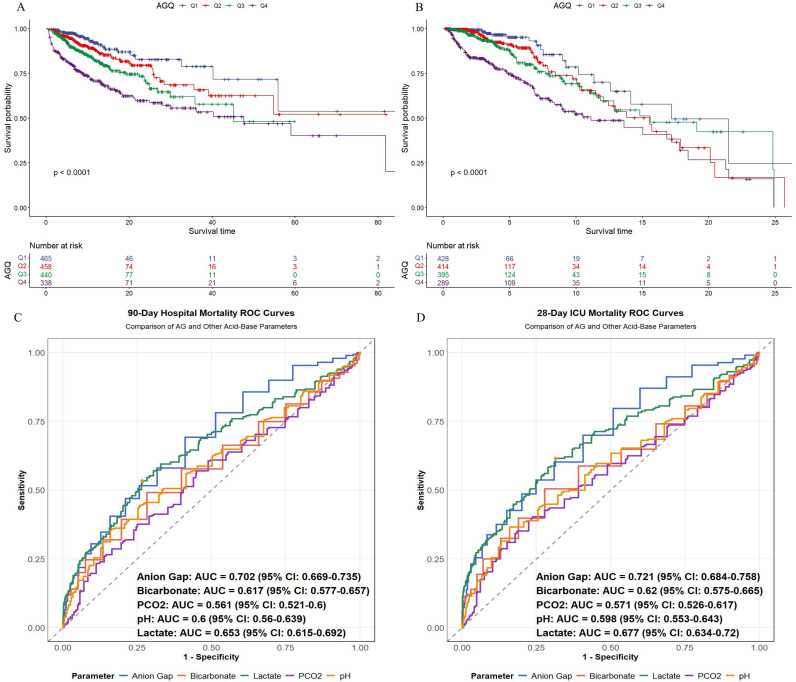
Kaplan–Meier survival analysis curves for all-cause mortality. Kaplan–Meier curves showing cumulative probability of all-cause mortality according to groups at 3 months (A), and 28 days (B); 90-Day Hospital Martality ROC Curve(C); 28-Day ICU Mortality ROC Curve(D).

Fig 2A and 2B display KM survival analysis curves based on serum AG quartiles to evaluate the incidence of major outcomes among the groups. Elevated serum AG values correlated with elevated risks of in-hospital and ICU mortality, with significant differences evident at both 28-day and 3-month intervals (Log-rank P < 0.0001). ROC analysis was conducted to further evaluate the predictive performance of serum AG in comparison to other acid-base balance indicators (Fig 2C and 2D). The results demonstrated that the AUC for serum AG in predicting 28-day ICU mortality was 0.721, while the AUC for predicting 3-month hospital mortality was 0.702, both significantly surpassing that of other acid-base balance indicators.

**Fig 3:**
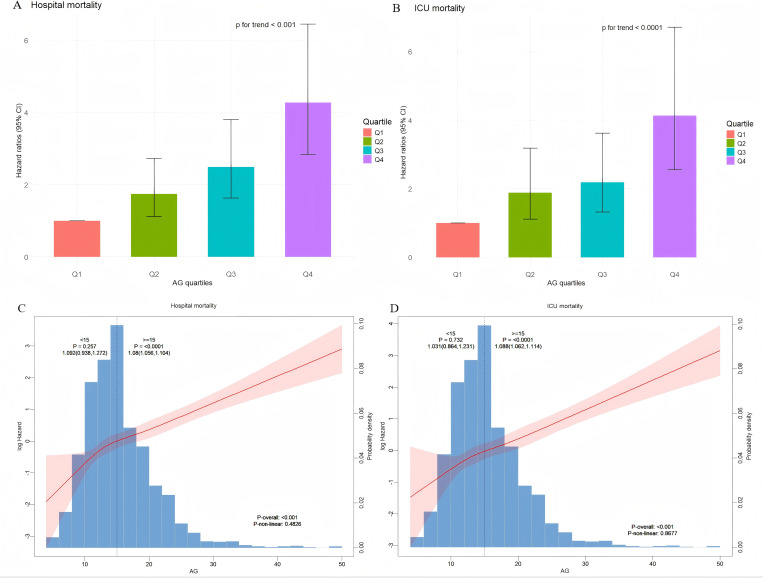
(A), (B): Hazard ratios (95% CIs) for hospital mortality and ICU mortality according to AG quartiles after adjusting for Sex, Age, BMI, Hypertension, Congestive heart failure, Moderate/Severe liver disease, Cerebrovascular disease, Metastatic solid tumor, PLT, RBC, WBC, BUN, Potassium, Sodium, GLU. Error bars indicate 95% CIs. The frst quartile is the reference. (C), (D): Restricted cubic spline curve for the AG hazard ratio. Heavy central lines represent the estimated adjusted hazard ratios, with shaded ribbons denoting 95% confdence intervals. (C) Restricted cubic spline for hospital mortality. (D) Restricted cubic spline for ICU mortality. HR, hazard ratio; CI, confdence interval; ICU, intensive care unit; AG, anion gap.

S3 Table presents binomial logistic regression results on all-cause mortality risk for critically ill patients with diabetic kidney disease (DKD), incorporating variables identified from univariable analyses (P < 0.05), as well as clinically recommended factors and prognostically relevant parameters based on physician experience. RDW, SOFA score, GCS score, and SpO₂ were identified as significant influencing variables. When serum AG was classified into quartiles, patients in the highest quartile (Q4) exhibited a 312% increased risk of all-cause mortality compared to those in the lowest quartile (Q1) [HR = 4.12 (95% CI: 2.66, 6.39)], following full adjustment for covariates, indicating a stepwise increasing trend with serum AG levels (Table 2; Fig 3A). Similar associations were confirmed in the multivariable Cox regression model analyzing ICU mortality (Table 2; Fig 3B). Additionally, RCS regression models showed a linear positive correlation between serum AG and both in-hospital and ICU mortality (P for non-linearity = 0.483 and P for non-linearity = 0.868) (Fig 3C and D).

**Table 2:** Cox proportional hazard ratios (HR) for all-cause mortality.

Character	Model 1		Model 2		Model 3	
	95%CI	P	95%CI	P	95%CI	P
Hospital mortality Continuous variable	1.09(1.07,1.11)	<0.0001	1.09(1.08,1.11)	<0.0001	1.09(1.07,1.11)	<0.0001
ICU mortality Continuous variable	1.08(1.07,1.10)	<0.0001	1.09(1.07,1.11)	<0.0001	1.10(1.07,1.12)	<0.0001
Hospital mortality quartile						
Q1	ref		ref		ref	
Q2	1.74(1.12,2.72)	0.01	1.81(1.16,2.83)	0.01	1.77(1.12,2.80)	0.01
Q3	2.49(1.63,3.80)	<0.0001	2.6(1.70,3.98)	<0.0001	2.46(1.58,3.82)	<0.0001
Q4	4.27(2.84,6.44)	<0.0001	4.59(3.04,6.92)	<0.0001	4.12(2.66,6.39)	<0.0001
p for trend		<0.0001		<0.0001		<0.0001
ICU mortality quartile						
Q1	ref		ref		ref	
Q2	1.89(1.12,3.18)	0.02	1.9(1.13,3.21)	0.02	1.84(1.08,3.15)	0.03
Q3	2.19(1.32,3.62)	0.002	2.28(1.38,3.77)	0.001	2.23(1.33,3.76)	0.003
Q4	4.14(2.55,6.71)	<0.0001	4.5(2.77,7.31)	<0.0001	4.48(2.66,7.54)	<0.0001
p for trend		<0.0001		<0.0001		<0.0001

Model 1: unadjusted

Model 2: adjusted for Sex, Age, BMI.

Model 3: adjusted for Sex, Age, BMI, Hypertension, Congestive heart failure, Moderate/Severe liver disease, Cerebrovascular disease, Metastatic solid tumor, PLT, RBC, WBC, BUN, Potassium, Sodium, GLU.

### Subgroup analysis

**Fig 4:**
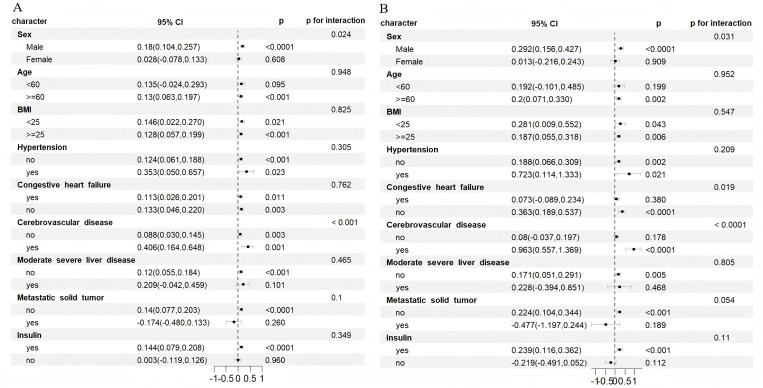
(A). Forest plots of hazard ratios for the hospital mortality in different subgroups. HR, hazard ratio; CI, confidence interval; BMI, body mass index. (B): Forest plots of hazard ratios for the ICU mortality in different subgroups. HR, hazard ratio; CI, confidence interval; BMI, body mass index.

Subgroup analysis was conducted to further explore the stratification value of serum AG for primary endpoints among different patient subgroups, encompassing sex, age, BMI, hypertension, congestive heart failure, cerebrovascular disease, moderate to severe liver disease, metastatic solid tumors and insulin use(Figs 4A and 4B). Serum AG was significantly associated with an increased risk of in-hospital mortality in the following DKD patient subgroups: male [HR = 0.18 (95% CI: 0.104, 0.257)], age ≥ 60 years [HR = 0.13 (95% CI: 0.063, 0.197)], BMI < 25 kg/m² [HR = 0.146 (95% CI: 0.022, 0.270)] and BMI ≥ 25 kg/m² [HR = 0.128 (95% CI: 0.057, 0.199)], with hypertension [HR = 0.353 (95% CI: 0.050, 0.657)] and without hypertension [HR = 0.124 (95% CI: 0.061, 0.188)], with congestive heart failure [HR = 0.133 (95% CI: 0.026, 0.201)] and without congestive heart failure [HR = 0.133 (95% CI: 0.046, 0.220)], with cerebrovascular disease [HR = 0.406 (95% CI: 0.164, 0.648)] and without cerebrovascular disease [HR = 0.088 (95% CI: 0.030, 0.145)], without moderate-to-severe liver disease [HR = 0.12 (95% CI: 0.055, 0.184)], without metastatic solid tumors [HR = 0.14 (95% CI: 0.077, 0.203)], and with insulin use [HR = 0.144 (95% CI: 0.079, 0.208)] (Fig 4A). In the ICU mortality stratification analysis, serum AG was significantly associated with male [HR = 0.292 (95% CI: 0.156, 0.427)], age ≥ 60 years [HR = 0.2 (95% CI: 0.071, 0.330)], BMI < 25 kg/m² [HR = 0.281 (95% CI: 0.009, 0.552)] and BMI ≥ 25 kg/m² [HR = 0.187 (95% CI: 0.055, 0.318)], without hypertension [HR = 0.188 (95% CI: 0.066, 0.309)], without congestive heart failure [HR = 0.363 (95% CI: 0.189, 0.537)], with cerebrovascular disease [HR = 0.963 (95% CI: 0.557, 1.369)], without moderate-to-severe liver disease [HR = 0.171 (95% CI: 0.051, 0.291)], without metastatic solid tumors [HR = 0.224 (95% CI: 0.104, 0.344)], and with insulin use [HR = 0.239 (95% CI: 0.116, 0.362)](Fig 4B). Additionally, interactions were observed in the gender and cerebrovascular disease subgroups for in-hospital mortality and in the gender, congestive heart failure, and cerebrovascular disease subgroups for ICU mortality (P for interaction < 0.05).

## Discussion

The association between serum AG and clinical outcomes was evaluated in a cohort of critically ill patients with DKD in the United States. Elevated AG was found to be associated with increased all-cause in-hospital and ICU mortality in this population. The significance of these associations was maintained following adjustment for potential confounding factors. Consequently, the serum AG levels may function as an independent prognostic indicator for critically ill DKD patients, thereby assisting clinicians in more effectively identifying high-risk patients and optimising management strategies during treatment decisions.

Clinically, AG can be calculated from serum or plasma electrolytes and is a routine measurement for hospitalized patients [24]. Elevated serum AG is attributable to either an excess production of acids or a diminished excretion of anions [25]. Numerous studies have investigated the association between serum AG levels and the prognosis or clinical outcomes of critically ill patients. For instance, Huang et al. reported that higher serum AG levels were associated with a greater risk of mortality in patients with acute kidney injury [26]. Additionally, Abramowitz et al.‘s study revealed that patients with early CKD had elevated serum AG levels, which were notably linked to all-cause mortality, even in cases with relatively preserved estimated glomerular filtration rate (eGFR) [27]. A substantial body of research has demonstrated the significant prognostic value of serum AG in a variety of patient populations with critical illnesses. For instance, Hu et al. identified a clear linear relationship among serum AG levels and both in-hospital and longer-term mortalities in disseminated intravascular coagulation (DIC) patients [28]. Lu et al. noted that an AG ≥ 15.12 mmol/L significantly increased the 30-day and 90-day death risk in AMI patients [29]. Notably, Jian et al. demonstrated that albumin-corrected AG (ACAG) had higher accuracy than traditional serum AG in predicting mortality in AMI patients, suggesting that albumin correction could further enhance the clinical utility of AG [30]. Additionally, Jhou et al. indicated that high serum AG levels were an independent prediction of in-hospital mortalities in acutely ischemic stroke patients [31]. These studies suggest that serum AG and its corrected forms may serve as important indicators of metabolic disturbances and patient prognosis across various diseases.

Nevertheless, the efficacy for serum AG in predicting death rates among critical care individuals has also been called into question by several studies. For instance, Rocktaeschel et al. reported that although serum AG can indicate the presence of hyperlactatemia, its capacity for predicting death among severely sick persons was relatively limited, with a ROC area under the curve (AUC) of 0.64 [32]. Additionally, Cusack et al. assessed serum AG and its corrected forms, such as strong ion gap (SIG), in a mixed medical-surgical critically ill patient population and found that the predictive ability of AG (AUROC = 0.59) was inferior to traditional indicators like pH and lactate (AUROC = 0.65) [33]. This is contrary to the results of our study. This discrepancy may stem from several factors. Firstly, the heterogeneity of study populations significantly impacts the predictive value of serum AG. These studies included a diverse range of critically ill patients who have experienced multiple complex and mixed metabolic acid-base disturbances, such as lactic acidosis, uremic acidosis, and hyperchloremic acidosis, potentially diminishing AG’s prognostic utility as a standalone metabolic marker. In contrast, the present study focused on DKD patients, wherein elevated serum AG primarily reflects chronic metabolic disorders associated with DKD and an increased unmeasured anion load, thereby demonstrating a more significant correlation with all-cause mortality prediction. Secondly, variations in the calculation and correction methods of serum AG across studies may contribute to inconsistent findings [34]. Traditional AG calculations often fail to adequately account for other strong ions, such as sodium and chloride, and serum albumin levels, which are commonly and significantly abnormal in critically ill patients. Consequently, uncorrected serum AG may underestimate its clinical significance under different pathological conditions. In contrast, this study employed multivariable adjusted models that comprehensively considered various confounding factors, including electrolyte balance, thereby more accurately assessing serum AG’s independent prognostic value. However, as albumin measurements were missing in over 80% of patient records, albumin was not incorporated as a covariate in the current analysis in order to minimize the potential for significant bias. Additionally, differences in study design and statistical methodologies are important factors contributing to the discrepancies in conclusions. Previous studies often had smaller sample sizes, potentially lacking the statistical power to detect serum AG’s subtle predictive role. Furthermore, the relationship between dynamic changes in serum AG and outcomes has not been adequately explored; future research incorporating the dynamic trends of serum AG may further elucidate its prognostic value.

The exact biological mechanisms that explain the link between serum AG and mortality in patients with DKD are still unclear. Prior research has shown that serum AG, as a critical indicator of acid-base balance, reflects alterations in unmeasured anions such as lactate, phosphate, and sulfate [35]. Ketone bodies are recognized as a significant class of unmeasured anions in patients with diabetes. In type 1 diabetes, characterized by absolute insulin deficiency, the incidence of diabetic ketoacidosis (DKA) is substantially increased, precipitating a rapid accumulation of ketone bodies; this buildup directly underlies the marked elevation of the anion gap (AG) and the associated rise in short-term mortality [36,37]. Furthermore, in the wider cohort of patients with diabetic kidney disease (DKD), chronic renal impairment—even in the absence of overt DKA—hinders the excretion of these acidic metabolites, resulting in a persistently elevated AG. DKD patients frequently experience metabolic acidosis, and an elevated serum AG signifies an increased acid load within the body, which may lead to multi-organ dysfunction and systemic inflammatory responses, thereby heightening the risk of mortality [38]. Furthermore, metabolic acidosis can disrupt calcium and phosphate metabolism, resulting in bone diseases and cardiovascular complications, which are significant contributors to the high mortality rates observed in DKD patients [39,40]. Additionally, an increased serum AG may indicate further deterioration of renal function, suggesting diminished renal excretion capacity and consequent accumulation of toxins. This accumulation not only exacerbates acid-base imbalances but also contributes to metabolic disorders and multi-system failure [41]. Elevated serum AG may also influence insulin resistance (IR), thereby participating in the progression of DKD. Metabolic acidosis has been shown to reduce the efficiency of insulin binding to its receptors and decrease the sensitivity of insulin signaling pathways, thereby aggravating IR. IR is a central pathological feature of diabetes and is closely associated with the progression of DKD. This pathological state accelerates glucose metabolism disorders and lipolysis, increasing endogenous acid load and creating a vicious cycle between metabolic acidosis and IR [42]. Moreover, IR-induced defects in insulin receptor signaling can provoke diabetic nephropathy-like pathological conditions even in the absence of hyperglycemia [43]. Additionally, Nakagawa et al. reported in animal models that IR reduces nitric oxide synthesis in glomerular endothelial cells, subsequently promoting vascular endothelial growth factor expression and enhancing macrophage infiltration, ultimately increasing mortality risk [44]. These combined physiological alterations play a role in initiating and advancing DKD, leading to unfavorable clinical outcomes. Therefore, an elevated AG not only serves as a marker of disease progression in DKD patients but may also directly or indirectly increase mortality risk through multiple interconnected mechanisms.

Although previous research has established a connection between serum AG, metabolic disturbances, and the prognosis of critically ill patients, its specific role on those with DKD has yet to be fully understood. By analyzing clinical data from DKD patients, it has been further confirmed that elevated serum AG not only reflects metabolic acidosis but is also closely associated with multiple organ dysfunction and an elevated risk of mortality. Within a particular group of critically ill patients with DKD, serum AG emerged as a standalone risk factor for heightened mortality. Moreover, given the prevalent and deadly nature of DKD, serum AG has proven effective in identifying high-risk patients. This discovery carries important clinical implications, facilitating early detection of at-risk individuals and potentially reducing the likelihood of major adverse outcomes.

The strengths of this work lie in its clarification of clinical importance of serum AG as an independent prognostic indicator in critically ill diabetic kidney disease (DKD) patients. However, several limitations are acknowledged. Firstly, the retrospective design of the study prevents the establishment of clear causal connections. Even after performing multivariate adjustments and subgroup analyses, there may still be residual confounding factors that can affect clinical outcomes. Secondly, only the serum AG levels at admission were utilized to assess their association with all-cause mortality in DKD patients, thereby lacking data on dynamic changes of serum AG during hospitalization and ICU stay. Dynamic monitoring of AG in clinical settings could provide greater predictive value. Lastly, due to the absence of albumin records, serum AG was not corrected for albumin, despite some studies indicating that hypoalbuminemia affects serum AG concentrations.

## Conclusions

In summary, the utility of serum AG has been extended to critically ill DKD patients, and it has been indicated that serum AG may serve as a potential indicator for stratifying mortality risk in DKD patients during hospitalization and ICU stays. Measuring serum AG levels is a vital aspect of clinical decision-making and disease management. Additional research is required to determine if managing serum AG levels can enhance clinical outcomes.

### Ethics approval and consent to participate

The Institutional Review Boards of the Massachusetts Institute of Technology and Beth Israel Deaconess Medical Center approved the use of the database for research purposes, granting a waiver of informed consent for studies utilizing the database. All procedures were conducted in accordance with applicable guidelines and regulations.

## Supporting information

Table S1 Characteristics and outcomes of participants categorized by AG. (DOCX)

Table S2 Characteristics and outcomes of participants categorized by Survivors and Non-survivors groups. (DOCX)

Table S3 Binary logistic regression analysis of the factors influencing all-cause death of the study population. (DOCX)

Fig S1 Distribution of anion gap (AG) stratified by mortality status for all-cause in-hospital death and ICU death, respectively. (JPG)
