# Supplementary material for: Association between serum anion gap and all-cause mortality in critically ill patients with diabetic kidney disease: Analysis of the MIMIC-IV database
Source: PLoS One. 2025 Aug 1;20(8):e0329269. doi: 10.1371/journal.pone.0329269 (PMC12316226; doi:10.1371/journal.pone.0329269)
Supplement: Table S1 — (DOCX) [file pone.0329269.s001.docx]

Table S1. Characteristics and outcomes of participants categorized by AG

| variable | Total (n=1716) | Q1-Q3 (n=1376) | Q4 (n=340) | statistic | p.value |
| --- | --- | --- | --- | --- | --- |
| Sex, n(%) |  |  |  | 0.89 | 0.34 |
| Female | 576(33.57) | 454(32.99) | 122(35.88) |  |  |
| Male | 1140(66.43) | 922(67.01) | 218(64.12) |  |  |
| Age, years,(median [IQR]) | 71.18(63.28,78.52) | 71.21(63.68,78.49) | 70.88(60.61,78.64) | 1.93 | 0.05 |
| BMI, kg/m^2^, n(median [IQR]) | 29.77(25.76,34.90) | 29.86(25.83,34.90) | 29.02(25.41,34.80) | 1.06 | 0.29 |
| Weight, kg, (median [IQR]) | 86.03(72.50,101.93) | 86.50(73.00,102.00) | 84.35(71.14,100.93) | 1.28 | 0.20 |
| Vital signs  SBP, mmhg, n(median [IQR]) | 115.15(107.50,126.08) | 115.59(108.25,126.50) | 113.27(103.10,123.30) | 2.69 | <0.01 |
| DBP, mmhg, n(median [IQR]) | 57.74(52.21,64.88) | 57.62(52.24,64.39) | 58.48(52.21,66.83) | -0.75 | 0.45 |
| TP, (median [IQR]) | 36.74(36.53,36.98) | 36.74(36.53,36.98) | 36.72(36.48,36.98) | 2.42 | 0.02 |
| SPO_2_, (median [IQR]) | 97.61(96.14,98.80) | 97.63(96.21,98.78) | 97.48(96.04,98.84) | 2.42 | 0.02 |
| HR, (median [IQR]) | 80.00(71.95,90.07) | 78.93(71.32,88.00) | 85.00(74.39,98.26) | -6.58 | <0.0001 |
| PCO_2_, (median [IQR]) | 45.00(40.00,50.05) | 46.00(41.00,51.00) | 41.00(36.00,47.00) | 7.92 | <0.0001 |
| PH, (median [IQR]) | 7.41(7.38,7.44) | 7.41(7.38,7.44) | 7.40(7.35,7.43) | 5.24 | <0.0001 |
| LAC, (median [IQR]) | 2.53(1.90,3.60) | 2.44(1.86,3.30) | 3.40(2.08,5.73) | -8.82 | <0.0001 |
| Medical scores  Sofa, (median [IQR]) | 6.00(4.00,9.00) | 6.00(4.00,8.00) | 8.00(6.00,11.00) | -11.87 | <0.0001 |
| ApsⅢ, (median [IQR]) | 51.00(39.00,65.00) | 47.00(38.00,60.00) | 65.00(52.00,83.00) | -13.26 | <0.0001 |
| SapsⅡ, (median [IQR]) | 42.00(35.00,52.00) | 41.00(34.00,49.00) | 51.00(40.00,63.00) | -10.86 | <0.0001 |
| GCS, (median [IQR]) | 15.00(14.00,15.00) | 15.00(14.00,15.00) | 15.00(14.00,15.00) | 0.54 | 0.59 |
| Commorbidities  Hypertension, n(%) |  |  |  | 8.10 | <0.01 |
| No | 1625(94.70) | 1292(93.90) | 333(97.94) |  |  |
| Yes | 91( 5.30) | 84( 6.10) | 7( 2.06) |  |  |
| Congestive  Heart failure, n(%) |  |  |  | 4.17 | 0.04 |
| No | 743(43.30) | 613(44.55) | 130(38.24) |  |  |
| Yes | 973(56.70) | 763(55.45) | 210(61.76) |  |  |
| Moderate/severe  Liver disease, n(%) |  |  |  | 5.17 | 0.02 |
| No | 1633(95.16) | 1318(95.78) | 315(92.65) |  |  |
| Yes | 83( 4.84) | 58( 4.22) | 25( 7.35) |  |  |
| Cerebrovascular disease, n(%) |  |  |  | 0.02 | 0.90 |
| No | 1442(84.03) | 1155(83.94) | 287(84.41) |  |  |
| Yes | 274(15.97) | 221(16.06) | 53(15.59) |  |  |
| Metastatic  Solid tumor, n(%) |  |  |  | 0.30 | 0.59 |
| No | 1675(97.61) | 1345(97.75) | 330(97.06) |  |  |
| Yes | 41( 2.39) | 31( 2.25) | 10( 2.94) |  |  |
| Medications  Insulin, n(%) |  |  |  | 2.15 | 0.14 |
| No | 65( 3.79) | 47( 3.42) | 18( 5.29) |  |  |
| Yes | 1651(96.21) | 1329(96.58) | 322(94.71) |  |  |
| Laboratory tests  HB, g/dL, (median [IQR]) | 9.70(8.70,10.80) | 9.70(8.70,10.90) | 9.60(8.58,10.60) | 1.79 | 0.07 |
| PLT, K/uL, (median [IQR]) | 181.00(137.00,235.00) | 180.00(139.00,232.05) | 190.00(125.00,246.00) | 0.39 | 0.70 |
| RBC, m/uL, (median [IQR]) | 3.31(2.94,3.74) | 3.32(2.95,3.75) | 3.26(2.88,3.67) | 1.99 | 0.05 |
| RDW, fL, (median [IQR]) | 15.10(14.10,16.70) | 14.90(13.90,16.40) | 16.06(14.80,17.90) | -8.17 | <0.0001 |
| WBC, K/uL, (median [IQR]) | 12.10(9.20,16.20) | 12.00(9.20,15.70) | 13.15(9.50,18.34) | -2.93 | <0.01 |
| AG, mEq/L, (median [IQR]) | 15.00(12.00,19.00) | 14.00(12.00,16.00) | 23.00(21.00,25.00) | -36.62 | <0.0001 |
| Bic, mEq/L, (median [IQR]) | 22.00(20.00,24.00) | 22.00(20.00,25.00) | 20.00(17.00,23.00) | 10.69 | <0.0001 |
| BUN, mg/dL, (median [IQR]) | 37.00(24.00,58.25) | 33.00(23.00,51.00) | 63.00(41.00,89.00) | -13.53 | <0.0001 |
| Cr, mg/dL, (median [IQR]) | 2.10(1.40,3.80) | 1.80(1.30,2.90) | 4.60(2.70,7.10) | -15.57 | <0.0001 |
| GLU, mg/dL, (median [IQR]) | 152.00(117.00,213.00) | 146.00(113.00,198.00) | 191.50(141.75,277.25) | -6.52 | <0.0001 |
| Sodium, mEq/L, (median [IQR]) | 138.00(136.00,141.00) | 138.00(136.00,141.00) | 138.00(135.00,141.00) | 0.86 | 0.39 |
| Potassium, mEq/L, (median [IQR]) | 4.60(4.20,5.10) | 4.60(4.10,5.00) | 4.80(4.30,5.40) | -4.56 | <0.0001 |
| Events  Los hospital  day | 10.11(6.36,17.38) | 9.96(6.51,15.98) | 11.09(5.77,21.01) | -2.06 | 0.04 |
| Los ICU  day | 3.12(1.48,6.22) | 2.93(1.40,5.70) | 4.05(1.96,7.96) | -2.75 | <0.01 |
| Hospital mortality |  |  |  | 85.64 | <0.0001 |
| Alive | 1434(83.57) | 1207(87.72) | 227(66.76) |  |  |
| Death | 282(16.43) | 169(12.28) | 113(33.24) |  |  |
| ICU mortality |  |  |  | 82.52 | <0.0001 |
| Alive | 1490(86.83) | 1246(90.55) | 244(71.76) |  |  |
| Death | 226(13.17) | 130( 9.45) | 96(28.24) |  |  |

Abbreviation: AG, Anion Gap; BMI, Body Mass Index; SBP, Systolic Blood Pressure; DBP, Diastolic Blood Pressure; TP, Temperature; HR, Heart Rate; LAC, Lactic Acid; SOFA, Sequential Organ Failure Assessment; APS3, Acute Physiology Score III; SAPSII, Simplified Acute Physiology Score II; GCS, Glasgow Coma Scale; HB, Hemoglobin; PLT, Platelet; RBC, Red Blood Cell; RDW, Red Cell Distribution Width; WBC, White Blood Cell; Bic, Bicarbonate; BUN, Blood Urea Nitrogen; Cr, Creatinine; GLU, Glucose.
