# Supplementary material for: Association between serum anion gap and all-cause mortality in critically ill patients with diabetic kidney disease: Analysis of the MIMIC-IV database
Source: PLoS One. 2025 Aug 1;20(8):e0329269. doi: 10.1371/journal.pone.0329269 (PMC12316226; doi:10.1371/journal.pone.0329269)
Supplement: Table S2 — (DOCX) [file pone.0329269.s002.docx]

Table S2 Characteristics and outcomes of participants categorized by Survivors and Non-survivors groups

| Variable | Total (n=1716) | Alive (n=1490) | Death (n=226) | Statistic | P value |
| --- | --- | --- | --- | --- | --- |
| Sex, n(%) |  |  |  | 0.16 | 0.69 |
| Female | 576(33.57) | 497(33.36) | 79(34.96) |  |  |
| Male | 1140(66.43) | 993(66.64) | 147(65.04) |  |  |
| Age, years,(median [IQR]) | 71.18(63.28,78.52) | 70.94(62.78,78.19) | 73.99(66.54,80.51) | -2.93 | <0.01 |
| BMI, kg/m^2^, n(median [IQR]) | 29.77(25.76,34.90) | 29.86(25.75,34.84) | 29.27(25.89,34.93) | -0.09 | 0.93 |
| Weight, kg, (median [IQR]) | 86.03(72.50,101.93) | 86.33(73.10,102.08) | 84.15(70.30,100.73) | 0.91 | 0.36 |
| Vital signs  SBP, mmhg, n(median [IQR]) | 115.15(107.50,126.08) | 115.80(108.08,126.49) | 111.02(102.21,122.08) | 4.85 | <0.0001 |
| DBP, mmhg, n(median [IQR]) | 57.75(52.21,64.88) | 57.86(52.26,64.95) | 57.48(52.13,64.38) | 1.39 | 0.16 |
| TP, (median [IQR]) | 36.74(36.53,36.98) | 36.74(36.53,36.96) | 36.73(36.44,37.16) | 1.06 | 0.29 |
| SPO_2_, (median [IQR]) | 97.61(96.14,98.80) | 97.67(96.28,98.80) | 96.86(95.31,98.72) | 3.85 | <0.001 |
| HR, (median [IQR]) | 80.00(71.95,90.07) | 79.40(71.86,88.66) | 86.16(73.92,100.56) | -5.29 | <0.0001 |
| PCO_2_, (median [IQR]) | 45.00(40.00,50.05) | 45.20(40.60,50.35) | 43.00(36.85,50.00) | 2.23 | 0.03 |
| PH, (median [IQR]) | 7.41(7.38,7.44) | 7.41(7.38,7.44) | 7.40(7.34,7.43) | 4.54 | <0.0001 |
| LAC, (median [IQR]) | 2.53(1.90,3.60) | 2.48(1.86,3.40) | 3.39(2.30,6.18) | -7.08 | <0.0001 |
| Medical scores  Sofa, (median [IQR]) | 6.00(4.00,9.00) | 6.00(4.00,8.00) | 9.00(6.00,12.00) | -9.38 | <0.0001 |
| ApsⅢ, (median [IQR]) | 51.00(39.00,65.00) | 48.00(38.00,62.00) | 67.00(53.25,86.75) | -12.01 | <0.0001 |
| SapsⅡ, (median [IQR]) | 42.00(35.00,52.00) | 41.00(34.00,50.00) | 53.00(43.00,66.00) | -10.44 | <0.0001 |
| GCS, (median [IQR]) | 15.00(14.00,15.00) | 15.00(14.00,15.00) | 15.00(14.00,15.00) | 1.88 | 0.06 |
| Commorbidities  Hypertension, n(%) |  |  |  | 0.63 | 0.43 |
| No | 1625(94.70) | 1408(94.50) | 217(96.02) |  |  |
| Yes | 91( 5.30) | 82( 5.50) | 9( 3.98) |  |  |
| Congestive heart failure, n(%) |  |  |  | 11.32 | <0.001 |
| No | 743(43.30) | 669(44.90) | 74(32.74) |  |  |
| Yes | 973(56.70) | 821(55.10) | 152(67.26) |  |  |
| Moderate/severe  liver disease, n(%) |  |  |  | 1.41 | 0.24 |
| No | 1633(95.16) | 1422(95.44) | 211(93.36) |  |  |
| Yes | 83( 4.84) | 68( 4.56) | 15( 6.64) |  |  |
| Cerebrovascular disease, n(%) |  |  |  | 10.23 | <0.01 |
| No | 1442(84.03) | 1269(85.17) | 173(76.55) |  |  |
| Yes | 274(15.97) | 221(14.83) | 53(23.45) |  |  |
| Metastatic  solid tumor, n(%) |  |  |  | 0.96 | 0.33 |
| No | 1675(97.61) | 1457(97.79) | 218(96.46) |  |  |
| Yes | 41( 2.39) | 33( 2.21) | 8( 3.54) |  |  |
| Medications  Insulin, n(%) |  |  |  | 11.17 | <0.001 |
| No | 65( 3.79) | 47( 3.15) | 18( 7.96) |  |  |
| Yes | 1651(96.21) | 1443(96.85) | 208(92.04) |  |  |
| Laboratory tests  HB, g/dL, (median [IQR]) | 9.70(8.70,10.80) | 9.68(8.70,10.80) | 9.72(8.60,10.90) | 0.24 | 0.81 |
| PLT, K/uL, (median [IQR]) | 181.00(137.00,235.00) | 179.00(137.00,231.85) | 200.00(137.00,259.75) | -1.56 | 0.12 |
| RBC, m/uL, (median [IQR]) | 3.31(2.93,3.74) | 3.31(2.94,3.73) | 3.33(2.89,3.77) | 0.25 | 0.80 |
| RDW, fL, (median [IQR]) | 15.10(14.10,16.70) | 15.00(14.00,16.50) | 15.90(14.60,17.70) | -5.78 | <0.0001 |
| WBC, K/uL, (median [IQR]) | 12.10(9.20,16.20) | 11.90 (9.10,15.60) | 14.55(10.33,19.50) | -3.97 | <0.0001 |
| AG, mEq/L, (median [IQR]) | 15.00(12.00,19.00) | 15.00(12.00,18.00) | 18.00(15.00,23.00) | -9.34 | <0.0001 |
| Bic, mEq/L, (median [IQR]) | 22.00(20.00,24.00) | 22.00(20.00,24.00) | 20.00(17.25,23.75) | 4.65 | <0.0001 |
| BUN, mg/dL, (median [IQR]) | 37.00(24.00,58.25) | 35.50(23.00,56.00) | 48.50(34.00,74.75) | -5.67 | <0.0001 |
| Cr, mg/dL, (median [IQR]) | 2.10(1.40,3.80) | 2.00(1.40,3.60) | 2.75(1.80,4.30) | -2.95 | <0.01 |
| GLU, mg/dL, (median [IQR]) | 152.00(117.00,213.00) | 148.00(115.00,202.75) | 196.00(142.00,278.75) | -5.46 | <0.0001 |
| Sodium, mEq/L, (median [IQR]) | 138.00(136.00,141.00) | 138.00(136.00,141.00) | 139.00(135.00,143.00) | -2.55 | 0.01 |
| Potassium, mEq/L, (median [IQR]) | 4.60(4.20,5.10) | 4.60(4.20,5.10) | 4.60(4.10,5.30) | -1.52 | 0.13 |

Abbreviation: AG, Anion Gap; BMI, Body Mass Index; SBP, Systolic Blood Pressure; DBP, Diastolic Blood Pressure; TP, Temperature; HR, Heart Rate; LAC, Lactic Acid; SOFA, Sequential Organ Failure Assessment; APS3, Acute Physiology Score III; SAPSII, Simplified Acute Physiology Score II; GCS, Glasgow Coma Scale; HB, Hemoglobin; PLT, Platelet; RBC, Red Blood Cell; RDW, Red Cell Distribution Width; WBC, White Blood Cell; Bic, Bicarbonate; BUN, Blood Urea Nitrogen; Cr, Creatinine; GLU, Glucose.
