# Supplementary material for: Association between serum anion gap and all-cause mortality in critically ill patients with diabetic kidney disease: Analysis of the MIMIC-IV database
Source: PLoS One. 2025 Aug 1;20(8):e0329269. doi: 10.1371/journal.pone.0329269 (PMC12316226; doi:10.1371/journal.pone.0329269)
Supplement: Table S3 — (DOCX) [file pone.0329269.s003.docx]

Table S3 Binary logistic regression analysis of the factors influencing all-cause death of the study population.

|  | Variable | OR | Lower  95%CI | Upper 95%CI | P value |
| --- | --- | --- | --- | --- | --- |
| (Intercept) | (Intercept) | 0.01321 | 3.32E-11 | 4403947 | 0.665987 |
| Age | age | 1.016148 | 0.998147 | 1.034738 | 0.080911 |
| Congestive heart failure | Congestive heart failure | 1.239636 | 0.867748 | 1.780755 | 0.24074 |
| Cerebrovascular  disease | Cerebrovascular  disease | 2.368084 | 1.596085 | 3.484939 | 1.46E-05 |
| RDW | RDW | 1.076245 | 1.004347 | 1.151476 | 0.034903 |
| WBC | WBC | 0.997765 | 0.984526 | 1.008834 | 0.703099 |
| AG | AG | 1.045387 | 1.002469 | 1.090388 | 0.038246 |
| Bic | Bic | 0.997812 | 0.955896 | 1.040906 | 0.919594 |
| BUN | BUN | 1.001741 | 0.995153 | 1.008192 | 0.599831 |
| Cr | Cr | 0.920764 | 0.832192 | 1.013141 | 0.09959 |
| PCO_2_ | PCO_2_ | 0.994269 | 0.97656 | 1.011815 | 0.52507 |
| PH | PH | 2.327048 | 0.201258 | 28.33177 | 0.500617 |
| LAC | LAC | 1.025035 | 0.959183 | 1.096066 | 0.466796 |
| GLU | GLU | 1.000291 | 0.999363 | 1.001195 | 0.471982 |
| Sodium | Sodium | 1.03215 | 1.000623 | 1.064703 | 0.045423 |
| Sofa | sofa | 1.080016 | 1.011579 | 1.153229 | 0.021172 |
| ApsⅢ | ApsⅢ | 1.033578 | 1.018401 | 1.049197 | 1.36E-05 |
| SapsⅡ | SapsⅡ | 1.007087 | 0.984209 | 1.0305 | 0.546604 |
| GCS | GCS | 1.120985 | 1.047671 | 1.201444 | 0.001063 |
| Insulin | Insulin | 0.532754 | 0.277894 | 1.066691 | 0.065347 |
| HR | HR | 1.005474 | 0.99502 | 1.015979 | 0.304077 |
| SBP | SBP | 0.998893 | 0.986815 | 1.010818 | 0.85644 |
| SPO_2_ | SPO_2_ | 0.853016 | 0.785818 | 0.924859 | 0.000134 |
| Male | Male | 1.032044 | 0.735542 | 1.457236 | 0.8563 |

Abbreviation: AG, Anion Gap; RDW, Red Cell Distribution Width; WBC, White Blood Cell; Bic, Bicarbonate; BUN, Blood Urea Nitrogen; Cr, Creatinine; LAC, Lactic Acid; GLU, Glucose; SOFA, Sequential Organ Failure Assessment; APS3, Acute Physiology Score III; SAPSII, Simplified Acute Physiology Score II; GCS, Glasgow Coma Scale; HR, Heart Rate; SBP, Systolic Blood Pressure;
