# Supplementary figures and images for: Association between serum anion gap and all-cause mortality in critically ill patients with diabetic kidney disease: Analysis of the MIMIC-IV database
Source: PLoS One. 2025 Aug 1;20(8):e0329269. doi: 10.1371/journal.pone.0329269 (PMC12316226; doi:10.1371/journal.pone.0329269)

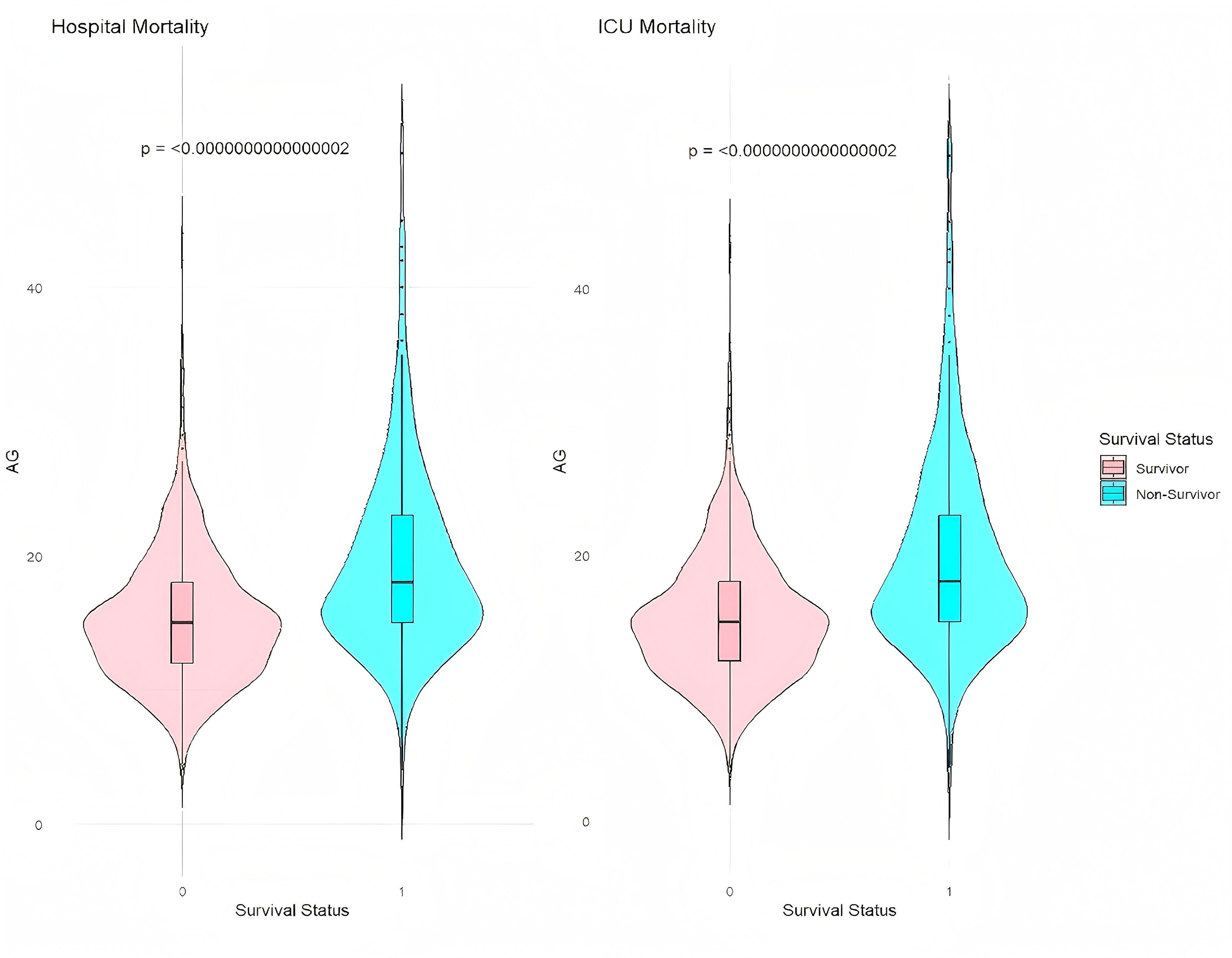

Supplement: Fig S1 — (JPG) [file pone.0329269.s004.jpg]
